# Disease burden in children with moderate to severe perennial allergic rhinitis and concomitant asthma in Canada, Denmark, and the United Kingdom

**DOI:** 10.1016/j.jacig.2025.100528

**Published:** 2025-07-01

**Authors:** Signe Voss Vahlkvist, Elana Lavine, Thomas Houmann Petersen, Mercedes Romano Rodriguez, Mark Aagren, Anne Sofie L. Loftager, Mette Bøgelund, Jose Alexandre da Graca da Maia e Costa

**Affiliations:** a Department of Paediatrics and Adolescent Medicine, Lillebaelt Hospital, University Hospital of Southern Denmark, Kolding, Denmark; b Department of Paediatrics, Humber River Hospital, Toronto, Ontario, Canada; c ALK-Abelló A/S, Hørsholm, Denmark; d EY Parthenon P/S, Frederiksberg, Denmark; e Nuffield Health Warwickshire Hospital, Royal Leamington Spa, Leamington Spa, United Kingdom

**Keywords:** Allergic rhinitis, perennial allergy, house dust mite allergy, burden of disease, caregiver burden, children, adolescents, health care resource use

## Abstract

**Background:**

Allergic rhinitis (AR) affects up to 40% of children in the United States and Europe. AR is often associated with asthma and has a negative impact on quality of life for the children and their families.

**Objective:**

We investigated the AR burden in children with moderate to severe perennial AR in Canada, Denmark, and the United Kingdom, focusing on the role of concomitant asthma. We assessed the health impact on the children, their receipt of allergy medication and health care services, and the impact on their families.

**Methods:**

An online survey was distributed to caregivers of children aged 5 to 17 with moderate to severe perennial AR (both with and without asthma) and to a control group of caregivers of children without allergies.

**Results:**

In total, 877 and 855 caregivers of children with perennial AR and without allergies, respectively, completed the survey. Children with AR and asthma, compared with those without asthma, experienced more sleep disturbances (69% vs 58%), schoolwork limitations (33% vs 22%), daily activities restrictions (55% vs 41%), and missed school hours (7.2 vs 4.6 hours per month). Children with AR and asthma had a higher receipt of allergy medication compared with those without asthma, and they also visited their general practitioner more often (4.6 vs 3.5 times a year). Overall, 32% of all caregivers of children with AR expressed dissatisfaction with allergy medication.

**Conclusion:**

Perennial AR, especially with concomitant asthma, imposes a substantial disease burden in children and their families, highlighting the need for long-term disease control.

Allergic rhinitis (AR) is a chronic inflammatory disorder affecting the nasal mucous membranes. The condition is mediated by IgE, which causes an inflammatory response in sensitized individuals.^1^, ^2^, ^3^ Epidemiologic studies have shown that AR affects approximately 20% to 30% of the population in both Europe and North America.^4^, ^5^, ^6^ Notably, children have a higher prevalence, with up to 40% of children and adolescents experiencing AR in the United States and Europe.^2^^,^^7^, ^8^, ^9^, ^10^, ^11^, ^12^

AR is associated with multiple comorbidities, including allergic conjunctivitis, atopic dermatitis, and allergic asthma.^13^, ^14^, ^15^ Particularly asthma is often linked to AR, and the co-occurrence of asthma can exacerbate symptoms in both disorders.^16^^,^^17^

Previous studies have established how poorly controlled AR among children hinders their daily activities and has a negative impact on their overall quality of life.^18^, ^19^, ^20^, ^21^ AR along with asthma in children can impair physical functioning and emotional well-being, and it can interfere with social interactions, learning abilities, and restorative sleep.^22^, ^23^, ^24^

When a child has severe AR, it negatively affects not only the child but also the entire family.^19^^,^^25^ A recent study has found that parents of children with allergic symptoms exhibited a significantly lower health-related quality of life compared with parents whose children did not experience such symptoms.^26^ Furthermore, AR has been associated with greater medication receipt and increased health care service utilization; studies indicate that individuals with AR may face double the medication costs and physician visits compared with individuals without AR.^27^, ^28^, ^29^ These facts highlight the widespread implications associated with this condition.

Little research exists on perennial AR in children, especially when concomitant asthma is present—despite the fact that they often coexist.^30^ Moreover, the majority of the existing literature on adults focuses either on perennial AR compared with seasonal AR or only on AR in general.^30^, ^31^, ^32^ This gap emphasizes the necessity of a more inclusive understanding of perennial AR’s and concomitant asthma’s impacts on children and their families in order to provide effective treatment strategies. Therefore, the aim of this cross-sectional study was to investigate the impact of moderate to severe perennial AR on children and their caregivers in Canada, Denmark, and the United Kingdom, with a specific focus on the impact of concomitant asthma.

## Methods

We conducted an online cross-sectional survey in Canada, Denmark, and the United Kingdom to investigate the impact of moderate to severe perennial AR and concomitant asthma on the daily lives of children and their families.

### Study and control population

Caregivers answered the survey about the relevant child, with inclusion criteria that required the child to be aged 5 to 17 years with caregiver-reported symptomatic, moderate to severe AR with at least one physician-diagnosed perennial allergy, no food allergy, and no previous or ongoing allergen immunotherapy (AIT) treatment. Severity and classification of AR were based on the Allergic Rhinitis and Its Impact on Asthma (ARIA) score. A matched control group of caregivers for children with no caregiver-reported allergies answered relevant sections of the survey. Caregivers in Canada and the United Kingdom with more than one child with perennial allergy were instructed to answer for the child whose allergy is worse.

### Data collection

Data were collected during January through April 2023 to assess the impact of perennial AR during the winter months. The data collection process was conducted differently in Denmark compared with the United Kingdom and Canada. In Denmark, all relevant caregivers were identified through Statistics Denmark via the Danish registers. The caregivers were contacted and invited to participate in the survey if their child had filled at least 2 prescriptions for allergy medication (antihistamine or intranasal corticosteroids) in the winter season (from October through February) in 2 consecutive years. In Canada and the United Kingdom, recruitment was carried out via Norstat’s email panels, which distributed the survey to a representative population. Norstat specializes in data collection for research, including market and health studies.^33^ The process for recruiting control participants mirrored that of the cases in the 3 different countries. To ensure a valid comparison, controls were paired with cases by matching age and sex.

Because this study was not a clinical trial, did not include direct contact with patients, and did not gather biological and/or human samples or identifiable personal information, Danish legislation does not require ethical review board approval.^34^ Before answering the survey, all caregivers gave consent to participate and provide information about their child and his or her allergy. Written informed consent to participate was not directly obtained but was inferred by completion of the questionnaire and by confirming the participants had read, understood, and accepted the purpose of the survey.

### Survey content and validation

The survey was developed through a multistage validation process. Initially, a focus group with individuals with long-term allergy was conducted to gain insights into living with AR. Furthermore, a comprehensive literature review and consultation with medical experts from Canada, Denmark, and the United Kingdom ensured the survey’s content was both relevant and applicable.

The study used the ARIA questionnaire, which has been validated in multiple studies for both AR and asthma.^35^ Before the final data collection, a pilot survey was conducted in order to make sure the respondents understood the questions and there were no technical errors, after which the survey was refined accordingly.

The final survey, consisting of 53 questions, was divided into 5 sections. The first section focused on allergy type, severity, symptoms, treatment history, and comorbidities. Medication receipt was also assessed in terms of type, frequency, and caregiver satisfaction. Caregiver satisfaction was measured from a very low extent to a very high extent. The second section focused on the burden of AR, assessing the level of trouble experienced by the child in their everyday life (eg, sleep quality, school attendance, and impact on activities), with a scale ranging from not troubled at all to extremely troubled. The third section assessed the child’s general child health using the generic Child Health Questionnaire alongside questions on health care service utilization. The fourth section consisted of the family impact of AR, including caregivers’ stress levels, family limitations, work absence, and household chores. Last, the fifth section consisted of questions related to socioeconomic factors.

### Statistical analysis

Survey responses were checked for errors and consistency before inclusion in the statistical analysis. The survey data underwent analysis through univariate descriptive statistics, including means (reported as results of the descriptive analyses), medians, frequencies, and standard deviation. Chi-square tests (for categorial variables) and *t* tests (for continuous variables) were performed to examine significant differences between groups. These tests, selected for their suitability in analyzing the types of data collected, are effective and intuitive for identifying significant patterns within subgroups. Significance was assessed at a level of *P* < .05. All analyses were conducted on complete case data, as the survey design required all relevant questions to be answered before submission, thus ensuring that no data were missing from the analyzed sample.

Data are presented for the full population, with additional subgroup analyses for the children with AR as well as for those with and without asthma. Because the allergy questions were not asked for the control population, differences were primarily assessed between children with and without asthma.

## Results

A total of 877 caregivers (315 in Canada, 251 in Denmark, and 311 in the United Kingdom) for a child with perennial AR, plus 855 caregivers (353 in Canada, 132 in Denmark, and 370 in the United Kingdom) for a child without any allergies, were included, with all caregivers answering on behalf of only one child. Table I provides the baseline characteristics of the 877 children with AR, and Table E1 (available in this article’s Online Repository at www.jaci-global.org) provides the baseline characteristics for the caregivers of children with AR. Table E2, also in the Online Repository, presents the demographics for the control population (both children and caregivers). Results are presented for all 3 countries combined.

**Table I:** Baseline demographics and characteristics of children with AR

	All (n = 877)	Asthma (n = 356)	No asthma (n = 521)	*P* values (asthma vs no asthma group)
**Biological sex, n (%)**				.600
Female	364 (42%)	144 (40%)	220 (42%)	
Male	513 (58%)	212 (60%)	301 (58%)	
**Age, years**				
Mean	11.6	11.5	11.7	.622
**Types of allergies, n (%)**				
Dust mites	797 (91%)	322 (90%)	475 (91%)	.716
Pollen	686 (78%)	298 (84%)	388 (74%)	.001
Animals	419 (48%)	214 (60%)	205 (39%)	<.001
Other year-round allergies	283 (32%)	133 (37%)	150 (29%)	.008
Oral allergy syndrome	101 (12%)	43 (12%)	58 (11%)	.666
**No. of allergies, n (%)**				<.001
1	82 (9%)	17 (5%)	65 (13%)	
2	330 (38%)	109 (31%)	221 (42%)	
3	327 (37%)	150 (42%)	177 (34%)	
≥4	138 (16%)	80 (22%)	58 (11%)	
**Comorbidities, n (%)**				
Asthma	356 (41%)	356 (100%)	0 (0%)	-
Eczema/atopic dermatitis	335 (38%)	149 (42%)	186 (36%)	.066
Wheezing	244 (28%)	162 (46%)	82 (16%)	<.001
Recurrent respiratory infection	211 (24%)	137 (38%)	74 (14%)	<.001
Dental malocclusion	187 (21%)	80 (22%)	107 (20%)	.492
ADHD or other mental illness	142 (16%)	66 (19%)	76 (15%)	.119
Nasal polyps	86 (10%)	37 (10%)	49 (9%)	.629
ASD	82 (9%)	41 (12%)	41 (8%)	.068
**No. of comorbidities, n (%)**				<.001
0	177 (20%)	0 (0%)	177 (34%)	
1	232 (26%)	61 (17%)	171 (33%)	
2	204 (23%)	100 (28%)	104 (20%)	
3	133 (15%)	86 (24%)	47 (9%)	
≥4	131 (15%)	109 (31%)	22 (4%)	
**Symptoms related to AR, n (%)**				
Stuffy nose, runny nose, sneezing, itchy nose, or postnasal drip	792 (90%)	316 (89%)	476 (91%)	.201
Itchy, red, swollen, sore, or watery eyes	600 (68%)	233 (65%)	367 (70%)	.118
Shortness of breath, chest tightness or pain, coughing, or wheezing	361 (41%)	238 (67%)	123 (24%)	<.001
Itchy skin reactions, skin pain, or redness of skin	386 (44%)	157 (44%)	229 (44%)	.966
**No. of weeks during a month the child is affected by their AR, n (%)**				.001
1-3 weeks	587 (67%)	215 (60%)	372 (71%)	
More than 3 weeks	290 (33%)	141 (40%)	149 (29%)	
**ARIA items, n (%)**				
The allergy is troublesome to the child	716 (82%)	287 (81%)	429 (82%)	.517
The allergy disturbs the child’s sleep	545 (62%)	244 (69%)	301 (58%)	.001
The allergy restricts the child’s daily activities (social life, sports, leisure)	407 (46%)	195 (55%)	212 (41%)	<.001
The allergy restricts the child’s participation in school or work	232 (26%)	116 (33%)	116 (22%)	.001
**Hours missed from class or school due to AR, mean**				
Mean number of hours	5.6	7.2	4.6	.001

Data are presented as nos. (%) unless otherwise indicated. Information was caregiver reported.

*ADHD,* Attention-deficit/hyperactivity disorder; *ASD,* autism spectrum disorder.

The most common perennial allergy was house dust mite (HDM) at 91%, and 41% of the children had concomitant asthma (Table I). The children with concomitant asthma had a higher number of both allergies and comorbidities compared with the children without asthma, whereas 55% and 13% of the children with and without asthma had 3 or more comorbidities, respectively, and 64% and 45% of the children with and without asthma had 3 or more allergies.

Caregivers for children both with and without asthma equally reported AR as troublesome for the child (81% and 82%). Compared with children without asthma, however, more children with concomitant asthma experienced sleep disturbance and were restricted in school participation and daily activities. They also missed more hours from class or school because of allergy-related problems: 7.2 hours on average during the previous month, compared with 4.6 hours on average for the children without asthma.

### Receipt of allergy medication and health care services

As Table II shows, about two thirds of the children with AR were prescribed 2 or 3 different types of allergy medication, with 76%, 74%, and 51% prescribed tablets or capsules, nasal sprays, and eye drops, respectively. The children with concomitant asthma were prescribed tablets or capsules and nasal sprays more frequently than the children without asthma. Receipt of allergy medication among the children with AR persisted for several months each year—an average of 7.1 months for tablets or capsules, 6.8 months for nasal sprays, and 5.2 months for eye drops—and all such time periods were longer for the children with concomitant asthma. In addition, the children aged 12 to 17 years received all 3 types of medication for longer periods compared with the children aged 5 to 11 years (see Table E3 in the Online Repository available at www.jaci-global.org).

**Table II:** Receipt of allergy medication

	All (n = 877)	Asthma (n = 356)	No asthma (n = 521)	*P* values (asthma vs no asthma group)
**No. of medications, n (%)**				.707
0	48 (5%)	17 (5%)	31 (6%)	
1	217 (25%)	88 (25%)	129 (25%)	
2	284 (32%)	111 (31%)	173 (33%)	
3	328 (37%)	140 (39%)	188 (36%)	
**Types of allergy medication, n (%)**				
Tablets or capsules	668 (76%)	270 (76%)	398 (76%)	.851
Nasal sprays	651 (74%)	282 (79%)	369 (71%)	.005
Eye drops	450 (51%)	178 (50%)	272 (52%)	.521
**Frequency of medication use, n (%)**				
Tablets or capsules				NA
Every day	267 (40%)	129 (48%)	138 (35%)	
2-6 times a week	162 (24%)	69 (26%)	93 (23%)	
Once a week	90 (13%)	34 (13%)	56 (14%)	
1-3 times a month	85 (13%)	18 (7%)	67 (17%)	
Less than once a month	52 (8%)	15 (6%)	37 (9%)	
I do not know	12 (2%)	5 (2%)	7 (2%)	
Nasal sprays				.043
Every day	238 (36%)	116 (41%)	122 (33%)	
2-6 times a week	171 (26%)	79 (28%)	92 (25%)	
Once a week	84 (13%)	25 (9%)	59 (16%)	
1-3 times a month	96 (15%)	36 (13%)	60 (16%)	
Less than once a month	50 (8%)	20 (7%)	30 (8%)	
I do not know	12 (2%)	6 (2%)	6 (2%)	
Eye drops				.257
Every day	73 (16%)	25 (14%)	48 (18%)	
2-6 times a week	123 (27%)	58 (33%)	65 (24%)	
Once a week	95 (21%)	38 (21%)	57 (21%)	
1-3 times a month	81 (18%)	30 (17%)	51 (19%)	
Less than once a month	64 (14%)	20 (11%)	44 (16%)	
I do not know	14 (3%)	7 (4%)	7 (3%)	
**Months per year when the child takes medication, mean**				
Tablets or capsules	7.1	8.0	6.6	<.001
Nasal sprays	6.8	7.4	6.4	.002
Eye drops	5.2	5.4	5.1	.507
**Days after AR flare-up when the child was affected, mean**				
Mean number of days	3.3	3.6	3.1	.408
**Did the child use more medication in the days following the AR flare-up?**				<.001
Yes	434 (49%)	216 (61%)	218 (42%)	
No	370 (42%)	113 (32%)	257 (49%)	
Don’t know	73 (8%)	27 (8%)	46 (9%)	
**Days after AR flare-up when the child used more medication than usual, mean**				
Mean number of days	3.6	4.1	3.2	.003

Data are presented as nos. (%) unless otherwise indicated. *NA,* Not applicable.

**Fig 1:**
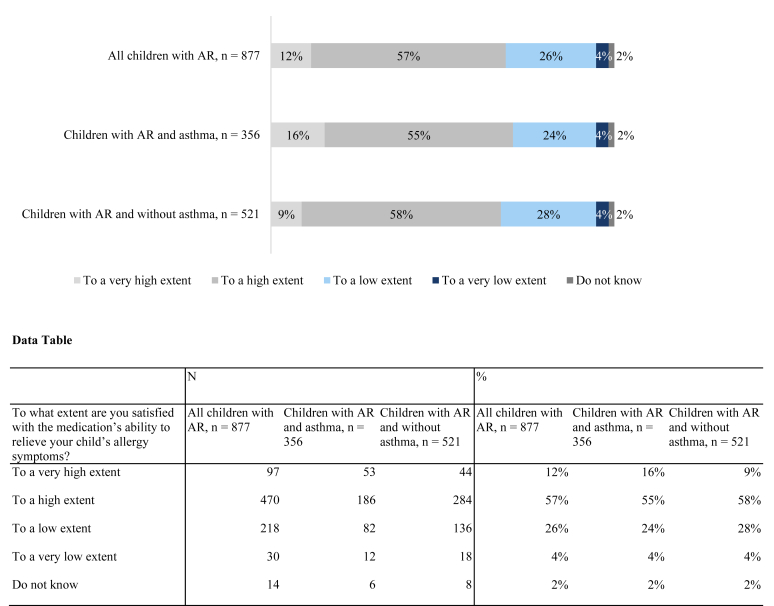
To what extent are you satisfied with the medication’s ability to relieve your child’s allergy symptoms?

Caregivers were also asked to assess the impact of the allergy medication using a scale of 0 (representing the poorest health) to 100 (representing optimal health) to indicate the child’s health when using the medication and when the medication is not available. The results were similar among the children with and without concomitant asthma, with caregivers rating the child’s health better while receiving the medication compared with when the child did not have access (74 vs 44). Furthermore, regarding satisfaction with the medication, the results in Fig 1 show that 69% of all caregivers of a child with AR were satisfied to a very high or high extent with the medication’s ability to relieve the child’s allergy symptoms. However, the data also show that one-third of caregivers were satisfied only to a low or very low extent with the allergy medication.

In terms of number of medications prescribed, no differences existed between the group that was satisfied to a high or very high extent and the group that was satisfied to a low or very low extent. However, the children in the group with low satisfaction were more affected by AR: 35% were affected 4 days a week or more and 40% more than 3 weeks a month, versus 17% and 30%, respectively, for the group with high satisfaction. The low satisfaction group also scored the child’s health lower on the 1-100 scale with access to medication compared with the high satisfaction group (64 vs 78).

Caregivers for a child with AR also rated statements about the child’s allergy medication (see Fig E1 in the Online Repository available at www.jaci-global.org), and more than half agreed or strongly agreed that the child’s health depends on the medication, that they sometimes worry about long-term effects of the medication, and that the medication prevents the child’s health from getting worse.

**Fig 2:**
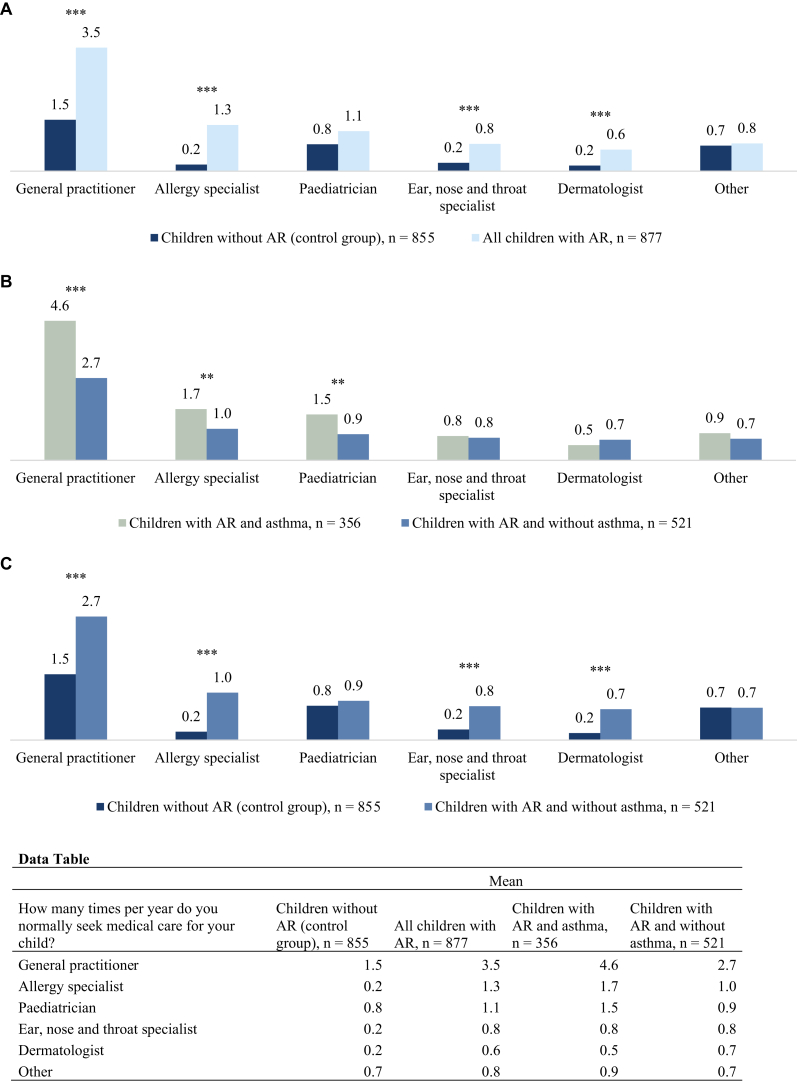
How many times per year do you normally seek medical care for your child? ∗*P* < .05, ∗∗*P* < .01, ∗∗∗*P* < .001.

Caregivers both for the children with AR and for those without were asked about the child’s use of health care resources. Fig 2, *A,* shows that caregivers for the children with AR sought medical care for the child more often than did caregivers for the children without allergy. This difference was significant (*P* < .001) for visits to general practitioners, allergy specialists, dermatologists, and ear, nose, and throat specialists. These results are highly driven by the children with AR and concomitant asthma, who sought medical care from a general practitioner, an allergy specialist, or a pediatrician significantly more often than the AR children without asthma and the children without allergy (*P* < .005) (Fig 2, *B* and *C*).

### Impact on caregivers and family

Table III shows that caregivers for a child with AR experienced feelings of stress and concern because of the child’s condition, with almost half reporting that the child’s AR has caused some, quite a bit, or a lot of stress. In addition, more than half (56-58%) expressed varying degrees of concern regarding the allergy’s impact on the child’s future education, work life, and social/family life (Table IV). A larger proportion of caregivers for a child with concomitant asthma reported feelings of stress and concern regarding their child’s future. However, caregivers for children aged 5 to 11 years were more concerned about the child’s future regarding education, work life, and social/family life compared with caregivers for children aged 12 to 17 years (Table E3).

**Table III:** Stress in caregivers and families due to AR

	All (n = 877)	Asthma (n = 356)	No asthma (n = 521)	*P* values (asthma vs no asthma group)
**Level of stress that AR causes in the caregiver, n (%)**				.007
A lot	75 (9%)	39 (11%)	36 (7%)	
Quite a bit	132 (15%)	69 (19%)	63 (12%)	
Some	158 (18%)	63 (18%)	95 (18%)	
A little bit	284 (32%)	105 (29%)	179 (34%)	
None at all	180 (21%)	64 (18%)	116 (22%)	
Not relevant	48 (5%)	16 (4%)	32 (6%)	
**Level of stress that AR causes in the caregiver’s partner, n (%)**				.023
A lot	32 (4%)	16 (4%)	16 (3%)	
Quite a bit	106 (12%)	54 (15%)	52 (10%)	
Some	154 (18%)	71 (20%)	83 (16%)	
A little bit	212 (24%)	85 (24%)	127 (24%)	
None at all	231 (26%)	79 (22%)	152 (29%)	
Not relevant	142 (16%)	51 (14%)	91 (17%)	
**Level of stress that AR causes in the family, n (%)**				.001
A lot	23 (3%)	14 (4%)	9 (2%)	
Quite a bit	82 (9%)	47 (13%)	35 (7%)	
Some	164 (19%)	76 (21%)	88 (17%)	
A little bit	242 (28%)	86 (24%)	156 (30%)	
None at all	266 (30%)	98 (28%)	168 (32%)	
Not relevant	100 (11%)	35 (10%)	65 (12%)	

Data are presented as nos. (%).

**Table IV:** Burden of AR among caregivers and families

	All (n = 877)	Asthma (n = 356)	No asthma (n = 521)	*P* values (asthma vs no asthma group)
**Caregiver’s concerns about the future, n (%)**				
Future work life				<.001
A lot	51 (6%)	28 (8%)	23 (4%)	
Quite a bit	98 (11%)	52 (15%)	46 (9%)	
Some	141 (16%)	63 (18%)	78 (15%)	
A little bit	220 (25%)	98 (28%)	122 (23%)	
None at all	367 (42%)	115 (32%)	252 (48%)	
Future social/family life				<.001
A lot	46 (5%)	20 (6%)	26 (5%)	
Quite a bit	106 (12%)	60 (17%)	46 (9%)	
Some	140 (16%)	63 (18%)	77 (15%)	
A little bit	219 (25%)	91 (26%)	128 (25%)	
None at all	366 (42%)	122 (34%)	244 (47%)	
Future education				<.001
A lot	73 (8%)	33 (9%)	40 (8%)	
Quite a bit	89 (10%)	52 (15%)	37 (7%)	
Some	122 (14%)	59 (17%)	63 (12%)	
A little bit	211 (24%)	92 (26%)	119 (23%)	
None at all	382 (44%)	120 (34%)	262 (50%)	
**How much AR has limited family activities, n (%)**				<.001
A lot	67 (8%)	34 (10%)	33 (6%)	
Quite a bit	87 (10%)	42 (12%)	45 (9%)	
Some	146 (17%)	70 (20%)	76 (15%)	
A little bit	239 (27%)	107 (30%)	132 (25%)	
None at all	338 (39%)	103 (29%)	235 (45%)	
**No. of caregivers who took time off from work due to AR in the past year, n (%)**				<.001
Yes	290 (33%)	164 (46%)	126 (24%)	
No	543 (62%)	168 (47%)	375 (72%)	
Don’t know	44 (5%)	24 (7%)	20 (4%)	
**Days absent from work in the past year due to AR, mean**				
Mean number of days	6.8	6.6	7.1	.704

Data are presented as nos. (%) unless otherwise indicated.

**Fig 3:**
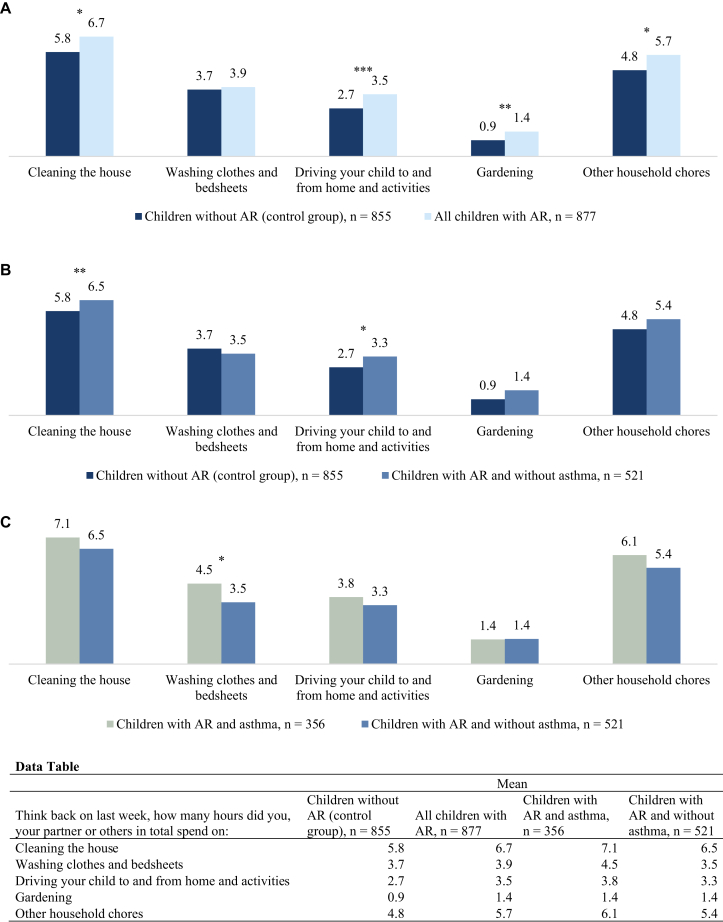
How many hours in total last week did you, your partner, or others spend on household chores? ∗*P* < .05, ∗∗*P* < .01, ∗∗∗*P* < .001.

Caregivers also experienced the impact in more practical aspects of their everyday lives. In the past year, 33% of caregivers took time off from work, for a mean of 6.8 days, because of the child’s AR. Caregivers of a child with concomitant asthma took time off from work more often compared with caregivers of a child without asthma (46% vs 24%). Furthermore, caregivers of a child with AR spent more time each week on household chores such as cleaning compared with caregivers of a child without allergies (Fig 3, *A*). Caregivers of a child with AR and concomitant asthma spent more time washing clothes and bedsheets compared with caregivers of a child with AR and without concomitant asthma (Fig 3, *B*). Caregivers of a child with AR and no concomitant asthma spent significantly more time cleaning the house and driving the child, compared with caregivers of a child without any allergies (Fig 3, *C*).

## Discussion

Our study of caregivers showed that moderate to severe perennial AR in children is associated with a considerable burden of disease—and an even bigger burden when concomitant asthma is present. In our multinational study population, the children were affected by both physical symptoms and restrictions in their leisure, school, and home activities. Children with perennial AR frequently required medication, often daily or multiple times a week, and children with concomitant asthma experienced even higher medication receipt throughout the year.

Our survey aimed to assess the overall satisfaction with current treatment standards for AR, not compare the effectiveness of different specific medication groups. Caregivers’ responses indicate that reported measures of children’s health were improved by taking the allergy medication. However, one third of the caregivers were satisfied with their current medication only to a very low or low extent. When comparing the low satisfaction group with the high satisfaction group, there was no difference in the number of different allergy medications prescribed. However, the group with low satisfaction was affected more days per week, as well as more weeks per month, compared with the group with high satisfaction. Additionally, caregivers in the group expressing low satisfaction with the current medication scored the child’s health lower, which indicates that the children in this group are treated with medication to the same extent as children in the high satisfaction group but may not experience the same effect of symptom relief.

Observational studies have reported inconsistent levels of satisfaction with allergy medications, contrasting with clinical research that demonstrates high efficacy. In a French study, 33% of children and adults perceived their symptomatic AR medication as unhelpful in the past month, and an Italian study indicated that more than half of patients were not satisfied with their treatment.^36^^,^^37^ In contrast, high satisfaction rates were reported in Finland, where 93% of patients undergoing HDM AIT were satisfied with their treatment, and a different French study indicated that 85% of children with AR and concomitant asthma were satisfied with AIT medication.^38^^,^^39^

Despite effective treatments and their frequent administration, dissatisfaction and a considerable disease burden remain. It is crucial for future research to delve into the reasons for this dissatisfaction by considering factors such as medication type, symptom specificity, and age differences. Additionally, studies often rely on physician-perceived patient satisfaction, which may not accurately reflect true patient sentiment, as health care providers tend to overestimate patient satisfaction.^29^^,^^38^ This underscores the need for general practitioners to recognize this matter and to implement regular follow-ups and personalized treatment adjustments.

Previous research has consistently indicated that AR in general is associated with increased utilization of health care services, encompassing both medication costs and visits to physicians.^27^, ^28^, ^29^ Our study confirmed that children with perennial AR sought medical care more often compared with children without AR, and that perennial AR impacts not only the child but also the entire family in terms of stress, worrying, and limitation in family activities. Thus, perennial AR in children has negative consequences for both the affected children and their families and regarding health care costs.

The co-occurrence of asthma intensified the challenges associated with AR, leading to multiple restrictions in the everyday life for children with AR, increased medication receipt, and an increase in caregiver anxiety. Our findings underscore the critical importance of adopting proactive measures to manage AR and minimize the risk of asthma persistence.^40^ In addition, our study showed a higher occurrence of recurrent respiratory infection among the children with perennial AR and concomitant asthma, which could be the reason for the increased medication receipt, health care resource use, and absence from school in this population.

From a policy perspective, our findings highlight a crucial need for treatments providing long-term symptom control in children with perennial AR and concomitant asthma. Effective allergy medication has the potential not only to improve the health and well-being of the affected children but also to alleviate the broader negative societal and familial impacts associated with the conditions. According to the caregivers participating in this study, the children are dependent of their allergy medication, which indicates that allergy medication serves an important role in managing AR. However, the results also indicate an unmet need in the field of children’s allergy medication. AIT is advised for the treatment of AR in individuals with disease that does not respond to corticosteroids or antihistamines, and clinical studies have shown that symptoms of both perennial AR and asthma can be reduced by AIT.^41^, ^42^, ^43^, ^44^, ^45^, ^46^ In both clinical trials and real-world studies, sublingual immunotherapy tablets have shown to be a safe and effective treatment in adolescents and children with HDM AR, indicating a promising long-term treatment solution for these age groups.^38^^,^^47^, ^48^, ^49^ For a deeper understanding, however, a more comprehensive evaluation of caregiver satisfaction with AIT is necessary.

### Strengths and limitations

This study has several strengths, including the large sample size of caregivers across 3 countries, which provides a comprehensive understanding of the impact of perennial AR and enhances the robustness of the findings. Using the Danish registers to identify the Danish population of children with AR (and the control group) ensures that these findings are based on a representative sample. The use of a control group is also a strength of this study, as it establishes a benchmark for the results and enriches understanding of the excess burden associated with perennial AR.

Limitations of our study include unavailable population registries for identifying the Canadian and United Kingdom populations, resulting in 2 different processes of recruitment. Selection bias is also a potential limitation as a result of the unknown characteristics of nonrespondents, which might affect the estimation of disease burden. However, the recruitment via the Danish registers and the email panels in Canada and the United Kingdom aimed to minimize this challenge, enhancing the reliability of the findings.

The multicenter approach of this study, while contributing to a larger and more diverse sample size, did not extend to a detailed analysis at each center level. This may have resulted in potential confounding factors specific to each local setting, such as allergen exposure, treatment approaches, health care access, and demographic factors. Another potential challenge is the timing of data collection in early 2023, which may coincide with coronavirus disease 2019 postlockdown infection, potentially amplifying the perceived burden of both AR and asthma. However, the alignment of our findings with existing research indicates that the reported burden in our study is unlikely to be solely a consequence of a postlockdown context.

Furthermore, the study’s cross-sectional and descriptive design captures data at a single point in time, which restricts the ability to establish causation. Another limitation of the study arises from the reliance on caregiver self-reports for perennial AR diagnoses. Furthermore, while caregiver reports can provide valuable insight into the experiences of children with AR, this approach also introduces potential biases in accurately representing the child’s experience.^21^^,^^50^

### Conclusion

Our study showed that the AR burden is high in children living with perennial AR, affecting several aspects of a child’s life, with implications for caregivers and immediate family. This impact is especially distinct for children with concomitant asthma. Increased medication receipt and increased health care resource use among these children, combined with the third of caregivers who express low satisfaction with the child’s current medication, emphasize a need for more effective long-term, disease-modifying treatment options.

## Disclosure statement

Supported by 10.13039/100031570ALK-Abelló A/S, Hørsholm.

Disclosure of potential conflict of interest: E. Lavine and J. A. d. G. d. Maia e Costa received fees from ALK-Abelló A/S for advisory services. S. V. Vahlkvist and T. H. Petersen received fees from ALK as coauthors of this study. M. R. Rodriguez and M. Aagren are employees of ALK-Abelló A/S. A. S. L. Loftager and M. Bøgelund are employees of EY Parthenon P/S, a paid vendor of ALK-Abelló A/S.

## Supplementary data

Supplementary Data

Supplementary Fig E1
