# Supplementary material for: Disease burden in children with moderate to severe perennial allergic rhinitis and concomitant asthma in Canada, Denmark, and the United Kingdom
Source: J Allergy Clin Immunol Glob. 2025 Jul 1;4(4):100528. doi: 10.1016/j.jacig.2025.100528 (PMC12444166; doi:10.1016/j.jacig.2025.100528)
Supplement: Supplementary Data [file mmc1.docx]

# Supplementary materials

**Supplementary Figure Legend:**

Figure E1: Caregivers indicate the extent to which they agree or disagree with statements about their child’s medication.
Note: Children with AR: N=877, children with AR and asthma N=356, and children with AR and without asthma N=521.

Table E1: Baseline demographics and characteristics of caregivers for children with AR

|  | Caregivers for a child with AR | | | |
| --- | --- | --- | --- | --- |
|  | All (n=877) | Asthma (n=356) | No asthma (n=521) | P-values (asthma vs no asthma group) |
| **Biological sex, n (%)** | | | | |
| Female | 623 (71%) | 253 (71%) | 370 (71%) | 0.987 |
| Male | 254 (29%) | 103 (29%) | 151 (29%) |  |
| **Age, years** | | | | |
| Mean | 42.2 | 41.9 | 42.4 | 0.374 |
| **Household income, n (%)** | | | | |
| UK | | | | |
| GBP 0-19,999 | 66 (21%) | 27 (20%) | 39 (22%) | NA |
| GBP 20,000-49,999 | 153 (49%) | 67 (50%) | 86 (49%) |  |
| GBP ≥ 50,000 | 81 (26%) | 37 (28%) | 44 (25%) |  |
| Unknown | 11 (4%) | 3 (2%) | 8 (5%) |  |
| Canada | | | | |
| CAD 0-19,999 | 23 (7%) | 6 (5%) | 17 (8%) | NA |
| CAD 20,000-49,999 | 75 (24%) | 28 (25%) | 47 (23%) |  |
| CAD ≥ 50,000 | 205 (65%) | 73 (66%) | 132 (65%) |  |
| Unknown | 12 (4%) | 4 (4%) | 8 (4%) |  |
| Denmark | | | | |
| DKK 0-299,999 | 22 (9%) | 9 (8%) | 13 (9%) | 0.414 |
| DKK 300,000-599,999 | 66 (26%) | 31 (28%) | 35 (25%) |  |
| DKK ≥ 600,000 | 135 (54%) | 55 (50%) | 80 (57%) |  |
| Unknown | 28 (11%) | 16 (14%) | 12 (9%) |  |

Abbreviations: AR: Allergic rhinitis, CAD: Canadian dollar, DKK: Danish krone, GBP: British pound sterling, n: number, NA: Not available.

Table E2: Baseline demographics and characteristics of the control group, both children and caregivers

|  | Children without AR (n=855) | Caregivers for a child without AR (n=855) |
| --- | --- | --- |
| **Biological sex, n (%)** | | |
| Female | 379 (44%) | 586 (69%) |
| Male | 476 (56%) | 269 (31%) |
| **Age, years** | | |
| Mean | 11.1 | 43.0 |
| **Age groups, n (%)** | | |
| 5-11 years | 456 (53%) | - |
| 12-17 years | 399 (47%) | - |
| **Household income, n (%)** | | |
| UK | | |
| GBP 0-19,999 | - | 75 (20%) |
| GBP 20,000-49,999 | - | 173 (47%) |
| GBP ≥ 50,000 | - | 110 (30%) |
| Unknown | - | 12 (3%) |
| Canada | | |
| CAD 0-19,999 | - | 24 (7%) |
| CAD 20,000-49,999 | - | 77 (22%) |
| CAD ≥ 50,000 | - | 241 (68%) |
| Unknown | - | 11 (3%) |
| Denmark | | |
| DKK 0-299,999 | - | 8 (6%) |
| DKK 300,000-599,999 | - | 28 (21%) |
| DKK ≥ 600,000 | - | 79 (60%) |
| Unknown | - | 17 (13%) |

Abbreviations: AR: Allergic rhinitis, CAD: Canadian dollar, DKK: Danish krone, GBP: British pound sterling, n: number.

Table E3: Use of allergy medication and caregiver’s concerns about the future, grouped by concomitant asthma and age

|  | Children with AR | | | | |
| --- | --- | --- | --- | --- | --- |
|  | Asthma (n=356) | No asthma (n=521) | 5-11 years (n=413) | 12-17 years (n=464) | P-values (5-11 years vs 12-17 years) |
| **Months per year when the child takes medication, mean** | | | | | |
| Tablets or capsules | 8.0 | 6.6 | 6.7 | 7.5 | 0.016 |
| Nasal sprays | 7.4 | 6.4 | 6.1 | 7.5 | <0.001 |
| Eye drops | 5.4 | 5.1 | 4.9 | 5.6 | 0.041 |
| **Caregiver’s concerns about the future, n (%)** | | | | | |
| Future work life | | | | | |
| A lot | 28 (8%) | 23 (4%) | 25 (6%) | 26 (6%) | 0.008 |
| Quite a bit | 52 (15%) | 46 (9%) | 56 (14%) | 42 (9%) |  |
| Some | 63 (18%) | 78 (15%) | 80 (19%) | 61 (13%) |  |
| A little bit | 98 (28%) | 122 (23%) | 98 (24%) | 122 (26%) |  |
| None at all | 115 (32%) | 252 (48%) | 154 (37%) | 213 (46%) |  |
| Future social/family life | | | | | |
| A lot | 20 (6%) | 26 (5%) | 25 (6%) | 21 (5%) | 0.002 |
| Quite a bit | 60 (17%) | 46 (9%) | 61 (15%) | 45 (10%) |  |
| Some | 63 (18%) | 77 (15%) | 79 (19%) | 61 (13%) |  |
| A little bit | 91 (26%) | 128 (25%) | 98 (24%) | 121 (26%) |  |
| None at all | 122 (34%) | 244 (47%) | 150 (36%) | 216 (47%) |  |
| Future education | | | | | |
| A lot | 33 (9%) | 40 (8%) | 44 (11%) | 29 (6%) | <0.001 |
| Quite a bit | 52 (15%) | 37 (7%) | 43 (10%) | 46 (10%) |  |
| Some | 59 (17%) | 63 (12%) | 76 (18%) | 46 (10%) |  |
| A little bit | 92 (26%) | 119 (23%) | 94 (23%) | 117 (25%) |  |
| None at all | 120 (34%) | 262 (50%) | 156 (38%) | 226 (49%) |  |

Abbreviations: AR: Allergic rhinitis, n: number, NA: Not available.
